# Cell/Tissue Microenvironment Engineering and Monitoring in Tissue Engineering, Regenerative Medicine, and In Vitro Tissue Models

**DOI:** 10.1155/2014/951626

**Published:** 2014-08-26

**Authors:** Nihal Engin Vrana, Vasif Hasirci, Garrett Brian McGuinness, Albana Ndreu-Halili

**Affiliations:** ^1^Protip SAS, 8 Place de l'Hôpital, 67000 Strasbourg, France; ^2^Institut National de la Santé et de la Recherche Médicale (INSERM), UMR-S 1121, “Biomatériaux et Bioingénierie”, 11 rue Humann, 67085 Strasbourg Cedex, France; ^3^Department of Biological Sciences and BIOMATEN, CoE Biomaterials and Tissue Engineering, Middle East Technical University, Biotechnology Research Unit Building, 06800 Ankara, Turkey; ^4^Mechanical and Manufacturing Engineering, Centre for Medical Engineering Research, Dublin City University, Dublin 9, Ireland; ^5^Department of Computer Engineering, Epoka University, Tirana 1039, Albania

In any engineered system, the understanding of the properties and interactions between the system components is of utmost importance for a successful outcome. The main components in engineered tissues are the cells, the materials used in construction of scaffolds, soluble or immobilized bioactive agents, and physical and chemical stimuli presented by the environment. As most of the mammalian tissues are constructed by bringing together repeating units of microscale complex tissue structures, understanding and control of all these components would provide the tissue engineers the capability to overcome clinical challenges as well as to develop technologies for high fidelity tissue models suitable for pharmacology, toxicology, and disease modelling applications.

As tissue engineering and regenerative medicine fields mature, the level of information about how cells interact with the surrounding scaffold materials and/or other cells has increased, too. Cell microenvironment, which can be defined as the sum of all the stimuli that stem from the neighborhood of a cell and have direct or indirect effects on a given cell, has become an important consideration in exerting more control over the interactions of the cells with engineered structures.

Another important aspect of tissue engineering and regenerative medicine is their temporal nature. An engineered tissue is actively remodelled over a period of time either in vitro or in vivo and currently our ability to influence this process in order to interfere with the sequence of events to achieve better regeneration is limited. However, developments in noninvasive monitoring methods and biosensor systems have begun to provide the necessary tools for tissue engineers to have real-time information about engineered tissues.

This special issue set out to demonstrate the current developments and future perspectives in the use of cell microenvironment engineering and monitoring in producing functional tissues. Several investigators contributed original research or review articles about the different aspects of cell microenvironment. M.-H. Yang et al. (2014) focused on a natural biomaterial, silk fibroin, and analyzed its interaction with fibroblasts. They used proteomic approaches to understand the interaction of the cells with the substrate formed by silk fibroin in the presence of carbon nanotubes. V. Cervelli et al. (2014) demonstrated the importance of the introduction of bioactive agents in the cell microenvironment in a clinical setting in the specific example of the treatment of male pattern hair loss. They have used platelet rich plasma (PRP) injections to the scalp and observed a significant increase in hair density, epithelial thickness, and number of hair follicles.

W.-Y. Lin et al. (2014) studied another important aspect of cell microenvironment, namely, the effect of dynamic mechanical stress/strain conditions. They have developed a system which can apply cyclic compressive stress to chondrocytes at physiologically relevant levels. They have shown that the application of stress has a direct effect on the chondrocyte metabolic activity and glycosaminoglycan secretion. Another aspect of cartilage tissue engineering, the extracellular matrix/chondrocyte interactions, was reviewed by Gao et al. (2014), where they focused on the signaling pathways that are active during chondrogenesis. In another review paper, Barthes et al. (2014) gave a comprehensive description of cell microenvironment and how each component can be used to direct cellular activity for tissue engineering applications, together with the current developments in the monitoring of artificial tissues.

The advances in tissue engineering not only established it as a field where solutions to serious clinical problems can be developed but also as a growing area where enabling technologies such as organ-on-a-chip systems for pharmacological purposes can be devised. In order to have models accurately mimicking artificial organs, it is important to have a good grasp of the specific microenvironments pertaining to each tissue type. We believe engineering of the cell microenvironment will be an important part of future tissue engineering activities.

*Nihal Engin Vrana* *Vasif Hasirci* *Garrett Brian McGuinness* *Albana Ndreu-Halili*

